# Supplementary material for: High-throughput sequencing approach for the identification of lncRNA biomarkers in hepatocellular carcinoma and revealing the effect of ZFAS1/miR-150-5p on hepatocellular carcinoma progression
Source: PeerJ. 2023 Feb 23;11:e14891. doi: 10.7717/peerj.14891 (PMC9968462; doi:10.7717/peerj.14891)
Supplement: Supplemental Information 1 [file peerj-11-14891-s001.doc]

Supplemental material 1. The basic characteristics of 18 hepatocellular carcinoma samples (adjacent tissues were provided by the same patient).

| Sample Num | Age (years) | Stage | Lymphatic Metastasis (Yes/No) | TNM |
| --- | --- | --- | --- | --- |
| LIHC-1 | 56 | II | No | N |
| LIHC-2 | 73 | III | No | N |
| LIHC-3 | 57 | III | No | N |
| LIHC-4 | 71 | I | No | N |
| LIHC-5 | 68 | II | No | N |
| LIHC-6 | 65 | III | No | T |
| LIHC-7 | 46 | I | No | N |
| LIHC-8 | 76 | II | No | T |
| LIHC-9 | 68 | III | No | M |
| LIHC-10 | 76 | III | No | M |
| LIHC-11 | 56 | I | No | T |
| LIHC-12 | 78 | I | No | N |
| LIHC-13 | 52 | II | No | T |
| LIHC-14 | 67 | III | No | M |
| LIHC-15 | 63 | I | No | N |
| LIHC-16 | 60 | III | No | M |
| LIHC-17 | 47 | / | No | / |
| LIHC-18 | 54 | III | No | M |

LIHC: Liver hepatocellular carcinoma; T-N-M: tumor-node-metastasis.
