# Supplementary material for: High-throughput sequencing approach for the identification of lncRNA biomarkers in hepatocellular carcinoma and revealing the effect of ZFAS1/miR-150-5p on hepatocellular carcinoma progression
Source: PeerJ. 2023 Feb 23;11:e14891. doi: 10.7717/peerj.14891 (PMC9968462; doi:10.7717/peerj.14891)
Supplement: Supplemental Information 2 [file peerj-11-14891-s002.docx]

Supplemental material 2. RNAs (lncRNA, miRNA and mRNA) sequencing information of 3 paired hepatocellular carcinoma and non-tumor tissues.

**LncRNA and mRNA**

| **sample** | **Raw reads** | **Raw bases** | **Clean reads** | **Clean bases** | **Error rate (%)** | **Q20(%)** | **Q30(%)** | **GC content (%)** | **rRNA Ratio (%)** |
| --- | --- | --- | --- | --- | --- | --- | --- | --- | --- |
| 1A | 1.22E+08 | 1.85E+10 | 1.22E+08 | 1.82E+10 | 0.025 | 98.07 | 94.05 | 45.65 | 5.09 |
| 1B | 1.05E+08 | 1.59E+10 | 1.05E+08 | 1.57E+10 | 0.0247 | 98.22 | 94.35 | 45.1 | 4.51 |
| 5A | 1.2E+08 | 1.81E+10 | 1.2E+08 | 1.77E+10 | 0.0247 | 98.19 | 94.45 | 50.05 | 4.24 |
| 5B | 1.09E+08 | 1.64E+10 | 1.09E+08 | 1.62E+10 | 0.0254 | 97.93 | 93.69 | 45.78 | 4.63 |
| 6A | 1.17E+08 | 1.77E+10 | 1.17E+08 | 1.74E+10 | 0.0247 | 98.18 | 94.38 | 46.65 | 5.56 |
| 6B | 1.03E+08 | 1.56E+10 | 1.03E+08 | 1.53E+10 | 0.0246 | 98.26 | 94.52 | 44.95 | 4.82 |

**MiRNA**

| **Sample** | **Raw reads** | **Adapter only** | **N reads** | **<18nt** | **>32nt** | **Clean reads** | **Error%** | **Q20%** | **Q30%** | **GC%** |
| --- | --- | --- | --- | --- | --- | --- | --- | --- | --- | --- |
| 1A | 2464538 | 4211 | 520 | 122750 | 161636 | 2175421 | 0.0448 | 89.44 | 83.05 | 68.31 |
| 1B | 17849283 | 4937 | 4342 | 519451 | 357147 | 16963406 | 0.0283 | 96.32 | 91.7 | 68.36 |
| 5A | 5721012 | 4669 | 1417 | 202666 | 278532 | 5233728 | 0.0307 | 95.33 | 89.86 | 68.32 |
| 5B | 10969971 | 4497 | 2630 | 144392 | 858612 | 9959840 | 0.0498 | 87.36 | 81.64 | 68.15 |
| 6A | 15852351 | 5563 | 4016 | 295651 | 263084 | 15284037 | 0.0292 | 96 | 90.92 | 68.24 |
| 6B | 19994906 | 6895 | 4792 | 884544 | 359726 | 18738949 | 0.0284 | 96.24 | 91.64 | 68.53 |

A: cancer tissue; B: control tissue.
