# Supplementary material for: High-throughput sequencing approach for the identification of lncRNA biomarkers in hepatocellular carcinoma and revealing the effect of ZFAS1/miR-150-5p on hepatocellular carcinoma progression
Source: PeerJ. 2023 Feb 23;11:e14891. doi: 10.7717/peerj.14891 (PMC9968462; doi:10.7717/peerj.14891)
Supplement: Supplemental Information 6 [file peerj-11-14891-s006.docx]

**Supplemental material 6.** RNAs in ceRNA.

| **RNAs** | **Details** |
| --- | --- |
| lncRNAs | ZFAS1, MZF1-AS1, SNHG10, FAM30A, AL024508.1, FBXL19-AS1, AC068473.5, AC074117.1 |
| miRNAs | miR-582-3p, miR-490-3p, miR-424-5p, miR-378i, miR-378c, miR-378a-3p, miR-150-5p, miR-139-5p, miR-96-5p |
| mRNAs | DCN,HGF,ARHGAP31,TIMP2,CELF2,ELN,MYLK,CD84,EVI5,MAST4,TGFBR3,SIDT1,P3H2,EFNB1,ESR1,SEMA6A,PTGS1,IL2RB,RNF125,RUBCNL,TFPI2,ITGB8,GATA3,CXCL12,HECA,LIFR,XRN1,CBLB,ST3GAL5,DNAJC16,OLFML3,CRYZ,SLC35A3,BCL11A,AMOT,CBFA2T3,SLC38A2,CALCOCO2,IRF4,FAM13A,CPNE8,MBNL2,FBLN5,CNKSR1,RGL1,RBMS3,OSBPL10,TMEM25,FLI1,CAMK4,SAMSN1,KCNMA1,ADAMTSL3,PALM2AKAP2,XDH,RCAN1,CYP3A4,CDC42EP3,PLEKHA2,RNF150,NAT1,MCC,BCL2,MLLT3,SUCLG2,NCKAP5,BASP1,HCLS1,TSPYL5,ZFP3,ZBTB20,PCDH9,ZFP90,NAP1L3,TPRG1,ASB13,ADH4,TRMT9B,NDST1,CDK16,BMF,NAT9,ALG2,C19orf25,LPGAT1,STK35,PAK4,ARHGEF11,SLC41A1,BEX2,PIGM,ABL2,ABT1,ARHGAP39,FADS1,LYSMD1,TMEM183A,STIP1,ZSCAN22,RABIF,CHML,PGR,ANTXR2,DOCK10,TRERF1,NMNAT1,MIEF1,ITGA4,FAM83D,PCDHGA8,LHX4,TRIM13,ZSWIM1,ZSWIM3,DACH1,STEAP3,CANT1,MASP1,ZBTB9,CSF1,HM13,ADGRG6,NRP2,TPK1,ABI3BP,IPCEF1,ERAP1,PCDHGA4,STEAP4,PRKAR2B,PRKD1,ZNF345,STX11,B2M,SEC22B,JAK2,CLDN11,POU2AF1,STON1,NAAA,CRYBG1,IVD,AGL,ENG,MYH10,CD274,SESN3,PKNOX1,SYTL2,FAT4,TXNIP,TEX14,GABARAPL1,SYNE1,ZCCHC3,MICALL1,CTTNBP2,SCAMP3,ADGRE5,TLR4,TTC39B,SVEP1,IL15,CLCN3,PLXNA4,UNC13D,CD163,KAZN,RANBP10,PBLD,GABBR1,ITPR1,COP1,KLF8,CEP131,KIF21B,TUFT1,ZNF462,PELI2,TOR3A,NYNRIN,SAT1,TLR1,LTBP4,CAD,PDE1C,PFAS,TNXB,C1QB,DYNC2H1,INPP4B,IL10RA,ENO2,GLRX,ITGA9,PTGS2,OSBPL5,NFAM1,ALDH8A1,CTPS2,DAAM2,AHDC1,ODR4,NDFIP2,MFAP3L,SLC39A8,HIVEP3,UST,NEK1,MYO1F,CCR7,GAB3,PRKCH,AAR2,SLC22A23,ZNF385A,AGXT2,ACSM2A,AKNA,RIOK1,TBRG1,NLRP1,TRMT61A,GIMAP8,P2RY13,EPB41L3,MRGBP,LAPTM4B,DISC1,ABCB4,CCDC86,NGFR,TIAM1,KCNK6,SND1,PROS1,FAR1,RNF135,CBR4,ADAMTS13,YY1AP1,TBC1D2B,SIAE,NEGR1,SHC1,FAM83H,PRKCB,TTLL7,RPS6KA1,SLC35D1,OBSL1,INTS7,TKFC,KIF26A,CDC37L1,PTMS,GPR137B,HDGF,MOCS2,SOX12,ZNF146,RASA3,ZDHHC11B,DHODH,LMBRD1,ADRB1,SCP2,PLOD3,NFATC1,IKZF1,PRUNE2,SMIM14,TMEM47,ARHGAP42,FAS,NUP62,ZNF792,SLC23A2,DGKE,SLC25A37,RNASEL,CYP4F11,WLS,PHGDH,RNF2,BMP6,ITPR3,KCNA3,PLCXD3,TMEM192,LY6E,DSN1,ZEB2,COL25A1,LZTFL1,LIMCH1,FILIP1,GNAO1,HDAC9,EPHA3,CACNB2,MYO9A,PDE10A,CCL22,VLDLR,IRAK3,FCMR, |
