# Supplementary figures and images for: High-throughput sequencing approach for the identification of lncRNA biomarkers in hepatocellular carcinoma and revealing the effect of ZFAS1/miR-150-5p on hepatocellular carcinoma progression
Source: PeerJ. 2023 Feb 23;11:e14891. doi: 10.7717/peerj.14891 (PMC9968462; doi:10.7717/peerj.14891)

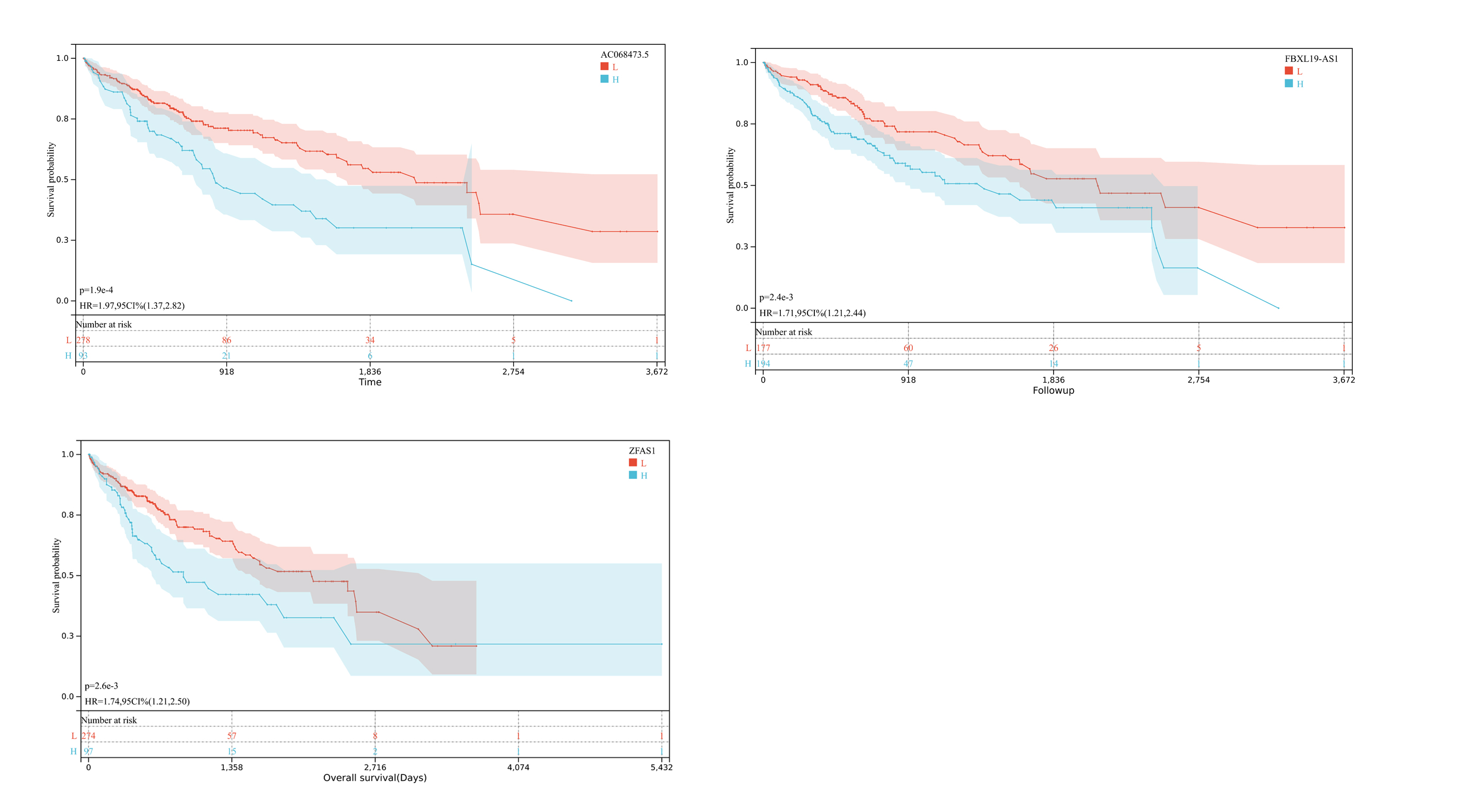

Supplement: Supplemental Information 8 [file peerj-11-14891-s008.jpg]
